# *Micrurus nigrocinctus* in Colombia: Integrating Venomics Research, Citizen Science, and Community Empowerment

**DOI:** 10.3390/toxins17060268

**Published:** 2025-05-27

**Authors:** Paola Rey-Suárez, Lina Preciado Rojo, Jeisson Gómez-Robles, Sanin Parra-Moreno, Erica Pachon-Camelo, Yirlys Fuentes-Florez, Bruno Lomonte, Julián Fernández, Mahmood Sasa, Vitelbina Núñez, Mónica Saldarriaga-Cordoba

**Affiliations:** 1 Grupo de Investigación en Toxinología, Alternativas Terapéuticas y Alimentarias, Facultad de Ciencias Farmacéuticas y Alimentarias, Universidad de Antioquia, Medellín 50010, Colombia; maria.preciado@udea.edu.co (L.P.R.); jeisson.gomez1@udea.edu.co (J.G.-R.); sanin.parra@udea.edu.co (S.P.-M.); erika-pachon@hotmail.com (E.P.-C.); vitelbina.nunez@udea.edu.co (V.N.); 2 Programa de Biología, Universidad de Antioquia, Medellín 50010, Colombia; yirlys.fuentes@udea.edu.co; 3 Instituto Clodomiro Picado, Facultad de Microbiología, Universidad de Costa Rica, San José 11501, Costa Rica; bruno.lomonte@ucr.ac.cr (B.L.); julian.fernandezulate@ucr.ac.cr (J.F.); mahmood.sasa@ucr.ac.cr (M.S.); 4 Museo de Zoología, Centro de Investigaciones en Biodiversidad y Ecología Tropical, Universidad de Costa Rica, San José 11501, Costa Rica; 5 Escuela de Microbiología, Universidad de Antioquia, Medellín 50010, Colombia; 6 Centro de Investigación en Recursos Naturales y Sustentabilidad (CIRENYS) and Escuela de Medicina Veterinaria, Universidad Bernardo O’Higgins, Santiago 8320000, Chile

**Keywords:** *Micrurus nigrocinctus*, venomics research, citizen science, coralsnake, community empowerment, Colombia

## Abstract

Snakebite is a high-priority neglected tropical disease, and a strategic goal based on four pillars has been recommended to reduce mortality and morbidity. One is empowering rural communities through citizen science, education, and engagement. In this study, an integrative approach was used to expand our knowledge of *Micrurus nigrocinctus* status and characterize its venom. Using citizen science data and field visits to local communities, 99 records of *M. nigrocinctus* distributed in Antioquia, Chocó, and Córdoba were obtained. Children, young people, and adults recognized *M. nigrocinctus* as the most common coral snake species in their region, and two specimens were recovered for venomic and Phylogenetic analyses. The *M. nigrocinctus* venom from Colombia exhibited similar chromatographic and electrophoretic profiles and biological activities and shared nearly identical protein families with Costa Rica. Commercial coral snake antivenoms also recognized and neutralized the whole venom from both countries. However, phylogenetic relationships showed greater divergence with specimens from Costa Rica. Involving communities helps prevent coral snake bites and facilitates access to rare specimens such as *M. nigrocinctus*, thereby enabling venom analyses, improving antivenom evaluation, and advancing toxinology research for medically significant species.

## 1. Introduction

Snakebite envenoming (SBE) represents a significant public health burden in many developing regions, particularly affecting resource-poor areas in sub-Saharan Africa, South Asia, Papua New Guinea, and Latin America. More than two million cases of SBE are reported annually, resulting in approximately 100,000 deaths and 300,000 cases of permanent disability [1]. Individuals at the highest risk live in rural areas, including children and young agricultural workers [2]. SBE occurs while people are engaged in everyday activities such as working in fields, collecting drinking water, sleeping in their homes at night, attending school, or even simply walking outdoors [3,4,5,6].

In 2017, the World Health Organization classified SBE as a high-priority neglected tropical disease [3,7], and in 2019, a strategic goal was established to reduce SBE-induced mortality and morbidity by 50% by 2030. This strategy is based on four pillars: (a) strengthening health systems, (b) ensuring safe and affordable access to quality antivenoms, (c) promoting research and innovation, and (d) community empowerment. According to WHO [8], empowering rural communities through citizen science, education, and engagement allows them to understand the associated risks better, adopt preventive measures, and seek timely and appropriate medical care, reducing the impact of snakebite [9,10]. By contributing their knowledge, communities not only manage to get involved in solving the problem that affects them but also participate in generating knowledge that promotes co-existence and the relationship between humans and snakes in these areas [8,11,12,13].

The involvement of local communities in a comprehensive approach to SBE could be key in situations where securing specimens for research programs or antivenom production is challenging. Such is the case of coral snakes (genus *Micrurus*), a group comprised of around 90 species [14], some of which are responsible for severe envenoming throughout the Americas [15,16,17]. Despite their medical relevance, knowledge about coral snake venoms remains limited, and the venoms of approximately two-thirds of the species remain uncharacterized. On the one hand, many species’ secretive habits and semi-fossorial lifestyle mean that knowledge about their ecology and distribution is highly fragmented [18,19]. This prevents a clear view of the species that may be present around communities in specific geographic areas and, therefore, the identification of those species of interest in the context of SBE [20]. Adding to these drawbacks is the scarcity of coral antivenoms, with only a few countries on the continent producing them [21]. This situation results in part from the difficulties in finding and capturing coral snake specimens but also from the restrictions on their maintenance in captivity [22] and the low venom yield that these elapids generally possess [23].

To reverse this situation, some antivenom research and production centers, such as the Clodomiro Picado Institute at the University of Costa Rica, are turning to local communities to secure specimens and identify sites where species of interest are relatively more abundant. These joint efforts have consolidated the maintenance of an important collection of *M. nigrocinctus*, a widely distributed species in the country whose venom is also used as an immunogen in the production of anticoral antivenoms for the Central American region [24]. This species is characterized by its relatively slender body and tricolor ring pattern and is distributed in Central and South America, from Mexico to northwestern Colombia [18]. Throughout this distribution, *M. nigrocinctus* is considered a locally abundant species responsible for most regional coral-related accidents [25]. However, it is not a snake that is easily collected, especially in the southern limit of its distribution in South America, so knowledge about its ecology and venom composition is mainly limited to populations in Costa Rica. *M. nigrocinctus* venom exhibits presynaptic neurotoxic effects, inducing a concentration-dependent depolarization in isolated preparations of mouse phrenic nerve-diaphragm, accompanied by ultrastructural changes in nerve terminals, probably due to the presynaptic action of PLA_2_ [26] and post-synaptic blockade of three-finger toxins [27]. Severe local myotoxicity in mice and morphological alterations in myogenic cells in vitro have also been reported for this venom [28,29]. The venom proteome of *M. nigrocinctus* from Costa Rica is mainly composed of phospholipases A_2_ (PLA_2_; 48%) and three-finger toxins (3FTx; 38%), along with proteins belonging to the ohanin (OH; 3.8%), L-amino acid oxidase (LAAO; 2.3%), C-type lectin (C-Lec; 2.2%), serine proteinase (SVSP; 0.7%), and nucleotidase (Nuc; 0.5%) families. Recent analyses with more sensitive techniques also identified other protein families [30].

Here, we employ an integrative approach to expand our knowledge of the status of *M. nigrocinctus* and characterize its venom at the southern limit of its distribution. Using citizen science data and visits to local communities in the Urabá and Chocó regions in Colombia, we re-evaluated the species’ known distribution in this country, analyzed its phylogenetic relationship with other coral snakes, characterized the biological activity and proteomic profile of its venom, and evaluated the ability of commercially available antivenoms to neutralize envenomation by this species. By including information on communities that coexist with *M. nigrocinctus*, our work also allows for an analysis of the perceptions of this and other coral snakes held by the people there. Importantly, our research provides practical insights into future research and public health strategies, enhancing our understanding of the species and its implications for public health.

## 2. Results

### 2.1. Distribution of M. nigrocinctus in Colombia Based on ‘Citizen Science’ and Scientific Database Reports

**Figure 1:**
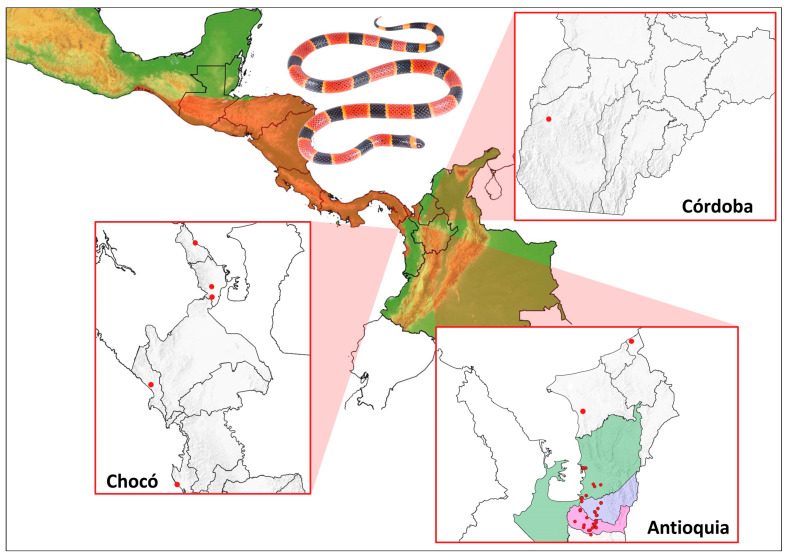
Distribution of *M. nigrocinctus* in Colombia based on ‘Citizen Science’ and scientific database reports. The species distribution is shaded in red. Red circles indicate recorded locations. In Antioquia, the municipalities with the most records were Apartadó (blue), Carepa (pink), and Turbo (green). The photograph shows an *M. nigrocinctus* specimen from Apartadó (Antioquia, Colombia).

**Figure 2:**
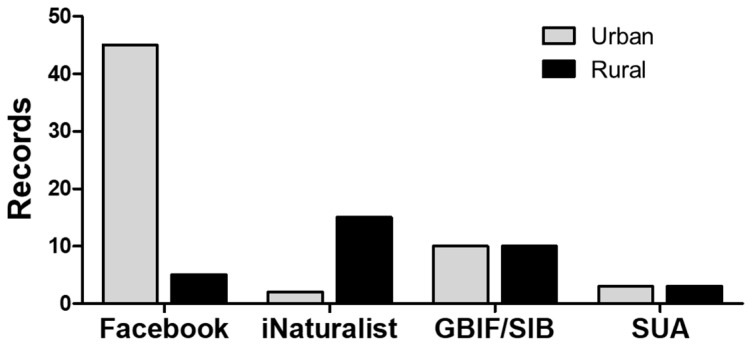
Reports for *M. nigrocinctus* in the Department of Antioquia based on ‘Citizen Science’ reports and scientific databases. GBIF/SIB: Biodiversity Information System/Biodiversity Information System; SUA: collection database of the Serpentarium of the University of Antioquia.

The data search and curation retrieved 99 records for *M. nigrocintus* (January 2018–February 2025), distributed across three departments (Supplementary Table S1). The highest number of records came from the Department of Antioquia, accounting for 94% (93 records). The municipalities with the most records were Apartadó (27 records), Carepa (34 records), and Turbo (28 records) (Figure 1). Five percent were from the department of Chocó, and a single record was for the department of Córdoba. The latter represents the first record of the species for this department (an individual deposited in the collection of the Serpentarium of the University of Antioquia—SUA (Supplementary Figure S1). Of the total records, only 31% (31 records) were obtained from specimens in collections or reported through Biodiversity Information Systems (GBIF or SIB). The community contributed the remaining 69% (68 records) through the reviewed platforms. Regarding the origin of the records, four out of the five records from Chocó came from rural areas, as did the only Córdoba Department record. In contrast, 60 (64%) of the *M. nigrocinctus* records in Antioquia were obtained from urban areas, of which 45 (75%) were obtained through different groups on the Facebook website, representing the total number of records obtained through this source (Figure 2).

According to the distribution of *M. nigrocinctus*, the largest number of recorded snakes are clustered in the municipalities of Apartadó, Carepa, and Turbo (Figure 1); therefore, educational activities were conducted in these locations. A total of 802 individuals participated in the educational workshops. Among them, 403 were children, 215 were young people, and 184 were adults. Within the adult group, 20 belonged to indigenous communities, 72 were university students from various disciplines, and 17 were farmers. Notably, 50 healthcare personnel from the hospitals of Apartadó and Carepa attended the sessions, along with 25 members of rescue teams from Apartadó.

These educational initiatives raised awareness about snakebite prevention, the importance of snake conservation, and appropriate first aid responses in case of a bite. While *M. nigrocinctus* was the focal species, including other regional species such as *Bothrops asper, Porthidium nasutum*, and *Micrurus camilae* added crucial context for understanding snakebite risks in the community. The community showed great curiosity about coral snakes, actively engaged with the information provided, and demonstrated the ability to identify the venomous snake species in the area. Healthcare personnel received clear guidance on the specific treatment for coral snake bites and instructions on the importance of rapidly transferring patients to higher-level hospitals in cases of envenomation presenting with early signs of respiratory paralysis.

Overall, participants in the educational activities recognized *M. nigrocinctus* as the most common coral snake species in their region. As a result of this engagement, two *M. nigrocinctus* specimens were recovered, one from Apartadó and one from Carepa. These individuals were subsequently used for molecular and venomic analyses.

### 2.2. Molecular and Phylogenetic Analysis of M. nigrocinctus from Colombia

**Figure 3:**
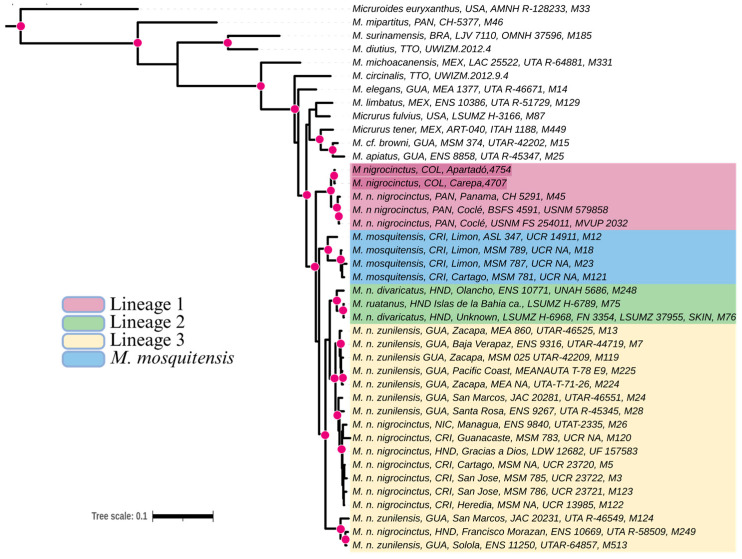
Phylogenetic tree resulting from Bayesian inference of ND4 and *Cytb* gene fragments from 41 coral snake species. Posterior probabilities (>0.95) are indicated by pink circles. Lineage conformations are shown according to [31].

The phylogenetic reconstruction reveals a closer relationship between the *M. nigrocinctus* samples from Colombia and Panama, forming a highly supported monophyletic group. This clade is sister to *M. mosquitensis* from Costa Rica, *M. ruatanus* from Honduras, and *M. nigrocinctus* from the rest of Central America (Figure 3).

### 2.3. Comparative Biochemical and Biological Characterization of M. nigrocinctus Venom from Colombia and Costa Rica

**Figure 4:**
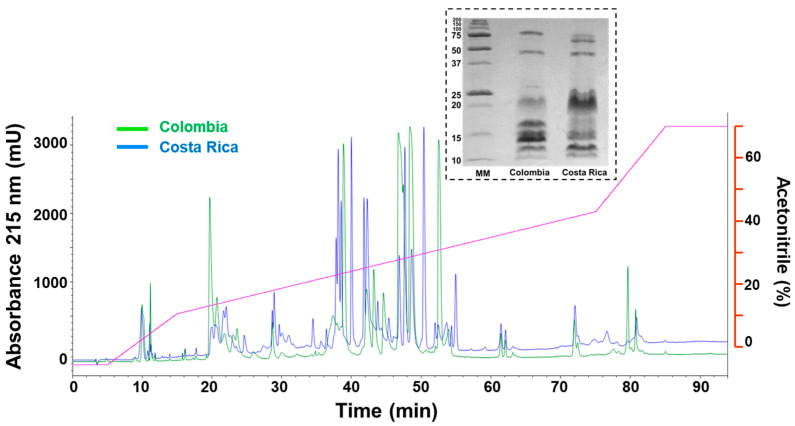
Comparison of the RP-HPLC profiles and SDS-PAGE (inset) of venom of *M. nigrocinctus* from Colombia (green) and Costa Rica (blue). Venom was fractionated on a C18 column and eluted with an acetonitrile gradient (purple line). The red axis corresponds to acetonitrile (%). For the SDS-PAGE, molecular weight markers are shown on the left in kDa.

The chromatographic profiles of *M. nigrocinctus* venoms from Colombia and Costa Rica were compared. Both venoms exhibited many prominent peaks eluting between 35 and 55 min. Notable differences were observed in the abundance of peaks eluting at 20 min and between 45 and 55 min when comparing both venoms (Figure 4). The comparative electrophoretic profiles for *M. nigrocintus* venoms from Colombia and Costa Rica showed abundant proteins in the region between 10 and 20 kDa, with notable band intensity differences indicating variations in the relative abundance of such small proteins. On the contrary, the band pattern for the region between 50 and 75 kDa was similar for both venoms (Figure 4).

**Figure 5:**
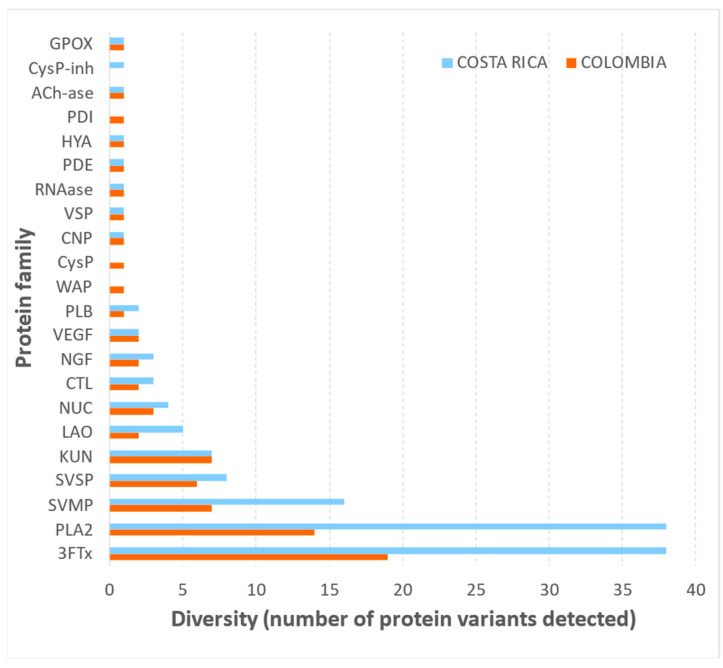
Diversity of proteins detected by shotgun proteomics in the venoms *Micrurus nigrocinctus* from Colombia and Costa Rica.

Using a shotgun proteomics approach, the venoms of *M. nigrocinctus* from Colombia and Costa Rica were compared, resulting in the detection of at least 75 and 134 proteins, respectively. Overall, both venoms presented almost the same protein families, except for a few minor components of the Cysteine proteinase (CysP) and Wapryn (Wap) families, which were not detected in the Costa Rican sample, and the CysP-inhibitor family, not detected in the Colombian sample (Figure 5; and Supplementary Table S2). Despite the conserved composition of these venoms in terms of protein families, the notable difference in the total number of proteins obtained in this comparison originates from the more significant number of variants belonging to the three-finger toxin (3FTx) and the phospholipase A_2_ (PLA_2_) protein families detected in the Costa Rican sample.

**Figure 6:**
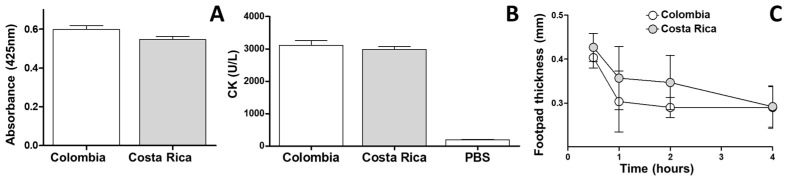
Comparative biological activities of *M. nigrocinctus* venom from Colombia and Costa Rica: (**A**) Phospholipase A_2_ activity upon the monodisperse synthetic substrate 4-nitro-3-octanoyloxy-benzoic acid (4-NOBA). (**B**) Myotoxic activity estimated by plasma creatine kinase (CK) activity. Each bar represents the mean ± SEM of triplicate assays. (**C**) Edema forming activity in the mouse footpad assay. Each point represents the mean ± SEM of triplicate assays. No statistically significant differences were found in any of the comparisons.

The PLA_2_, myotoxic, and edema-forming activity of both venoms were comparable since statistically significant differences were not observed (*p* > 0.05) (Figure 6).

The intraperitoneal (i.p.) LD_50_ for *M. nigrocinctus* venom from Colombia was estimated at 8.6 μg/mouse (95% CI = 3.18 to 15.8), equivalent to 0.5 μg/g of body weight.

### 2.4. Immunorecognition and Neutralization by Commercial Coral Antivenoms

**Figure 7:**
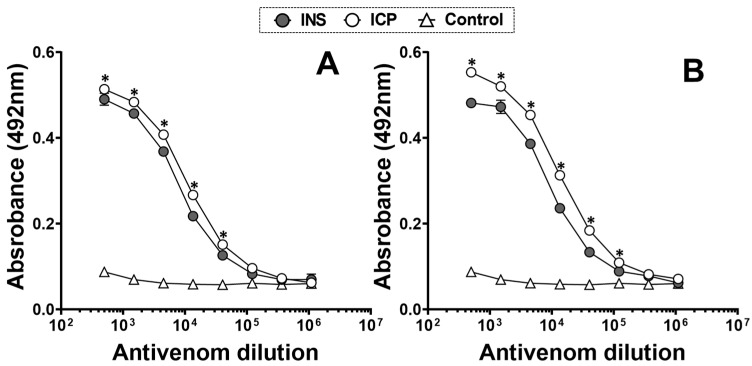
Recognition of commercial coral antivenoms against *M. nigrocinctus* venom from (**A**) Colombia and (**B**) Costa Rica. Each point on the graph represents the mean ± SD of the triplicate. * (*p* < 0.05) indicates a significant difference between both venoms.

Commercial coral antivenoms demonstrated a clear recognition of whole *M. nigrocinctus* venoms from Colombia and Costa Rica when antibody titers were evaluated by ELISA. The anticoral-ICP antivenom exhibited a slightly stronger recognition of both venoms compared to the anticoral-INS antivenom, which was statistically significant (*p* < 0.05; Figure 7A), with the highest binding observed for *M. nigrocinctus* venom from Costa Rica, which is used in its production (Figure 7B).

In addition, by preincubation assay, the anticoral-ICP antivenom neutralized the lethal effect of *M. nigrocinctus* venom from Colombia in a proportion of 0.2 mg/mL. In contrast, anticoral-INS partially neutralized the lethal effect of this venom when tested at the same proportion (Table 1).

## 3. Discussion

In this study, we aimed to increase knowledge on the poorly studied population of *M. nigrocinctus* in Colombia by integrating information gathered from biological collections, as well as communities regarding its occurrence in different geographical areas, with data obtained from laboratory analyses on its venom characteristics such as protein composition, toxic activities, neutralization by commercially available antivenoms, and the phylogenetic relationships of this population with other coral snakes.

Data related to specimens preserved in biological collections constitutes a valuable source of information in terms of morphology, diet, reproduction, and distribution, among various other aspects [32,33]. Biological collections contain fundamental data that allow us to fill the gaps around these species [31,33,34,35]. However, elusive organisms that are difficult to find in the field, such as snakes of the *Micrurus* genus, often have significant gaps in many aspects mentioned above [19]. For these specimens, a helpful way to collect biological data is the information provided for local communities, such as photographs, information about geographic location, and date–time information [36]. In addition, developing and enhancing ‘citizen science’ platforms can potentially reduce the shortfall of information related to *Micrurus* species.

The distribution of *M. nigrocinctus* in Colombia has been thus far limited to the northern Chocó biogeographic region [20,37]. These records are associated with the departments of Chocó and Antioquia. Although the area of Antioquia with the highest number of records of *M. nigrocinctus* borders the department of Córdoba, to date, there were no records of the species for this department, to the best of our knowledge. Different studies have reported the presence of other species of coral snakes, such as *M. dissoleucus*, *M. dumerilii,* and *M. mipartitus,* in various areas of Córdoba [38,39,40]. Therefore, the record presented here constitutes the first report in the literature on species distribution for the Department of Córdoba.

Most reports of *M. nigrocinctus* in Colombia occurred in urban areas, indicating relatively frequent encounters between humans and coral snakes in such settings, similar to the occurrence in Costa Rica [41,42]. This epidemiological pattern has been observed in studies of coral snakebite incidence, where more than half of the events occurred in urban areas, contrasting with the pattern of viper bites [43]. It is known that some coral snake species in the Amazon, such as *M. surinamensis* and *M. lemniscatus*, live near or within urban perimeters, in fragments of vegetation, or even close to residential settlements [44], which increases the likelihood of snakebite incidents in these areas.

Thanks to this articulation with communities in regions with abundant *M. nigrocinctus*, several specimens were collected to determine phylogenetic relationships with other *Micrurus* species. Over time, the evolutionary relationships within the *Micrurus* genus, as well as species delimitations, have been complicated and unclear [45,46] because most systematic evaluations of *Micrurus* have been heavily based on phenotypic traits (e.g., coloration, scalation, hemipenis morphology, etc. [18]. Meanwhile, there is a limited number of molecular studies, which are restricted mainly in terms of the number of species and/or geographic sampling [47,48,49,50,51]. This has incorrectly classified many regional color variants as distinct species or subspecies within the genus [52]. Therefore, addressing species diversity within the *Micrurus* genus remains of vital importance from biological, medical, and conservation perspectives [51,52].

Recent studies have described *Micrurus nigrocinctus* as a species complex due to its taxonomic diversity, forming polyphyletic groups that include species such as *M. mosquitensis*, *M. ruatanus*, and *M. nigrocinctus* from several localities across Central America [31]. These authors emphasize that the complex comprises at least three distinct lineages at the species level. In this way, the phylogenetic analysis of this study revealed a well-supported clade formed by *M. nigrocinctus* from Colombia and Panama, suggesting that *M. nigrocinctus* from Colombia is part of lineage 1. This clade is monophyletic and highly divergent from all other *M. nigrocinctus* lineages from Central America and, until now, has been restricted exclusively to Panama [31].

The proteomic profiling of *M. nigrocinctus* venom from Colombia showed a qualitatively similar protein family composition when compared to venom from Costa Rica, and the identified components are in general agreement with previous studies on the proteome of the latter venom [29,30]. However, the notable difference observed in the diversity of protein variants for 3FTx and PLA_2_ components between both venoms is intriguing and would demand further studies to establish the underlying mechanisms. Our findings suggest the possibility that a larger array of genes for these toxins could be either (a) present, (b) expressed, or (c) differentially regulated in the Costa Rican specimens, as compared to Colombian counterparts. Since our proteomic analyses were conducted using a general database for snakes (Uniprot Serpentes), future studies should address this question by complementing the proteomic data with specific venom gland transcriptomic data of *M. nigrocinctus*, which are currently unavailable.

The venom of Costa Rican *M. nigrocinctus* is known to be PLA₂-rich in its composition [29] and has demonstrated strong local myotoxic activity in the mouse model [28,53,54]. In this context, the venom of *M. nigrocinctus* from Colombia exhibited PLA₂ activity (4-NOBA), edema-forming, and myotoxic activities similar to the Costa Rican venom of this species. Likewise, the LD₅₀ of *M. nigrocinctus* from Colombia was similar to that reported for the Costa Rican population [22,24], *M. mosquitensis* [55], and *M. ruatanus* [56] —species that belong to the *M. nigrocinctus* complex—further supporting the phylogenetic relationships described within this group.

As shown by the ELISA titration curves, the anticoral-ICP antivenom exhibited somewhat stronger recognition of *M. nigrocinctus* venom from Colombia, resulting in antibody binding signals nearly as high as those obtained for the homologous venom (*M. nigrocinctus* from Costa Rica) used in its production. The results highlight the high degree of antigenic conservation between the venoms from the two populations. On the other hand, the anticoral-INS antivenom showed good cross-recognition of *M. nigrocinctus* venom from Colombia despite this venom not being included in the immunizing mixture for its production [57]. Our findings align with previous studies, which observed cross-recognition of *M. nigrocinctus* and *M. mosquitensis* venoms from Costa Rica by the anticoral-INS antivenom [58].

The observed immunorecognition and neutralization are likely due to the antigenic similarity reported for venoms that exhibit a PLA_2_-predominant phenotype. It has been previously noted that coral snake venoms with a PLA_2_ predominance are better recognized and neutralized by ICP antivenom than those dominated by a high proportion of 3FTx proteins [55,59,60,61]. Thus, the dichotomous grouping of *Micrurus* venoms according to their relative abundance of 3FTx and PLA_2_ protein families is not only a quantitative feature but also a qualitative one by possibly reflecting the antigenic divergence of proteoforms between the two groups [30]. Nevertheless, it has been shown that the ICP antivenom can neutralize the venom of *M. ruatanus*, a highly lethal species with 3FTx predominance [56] that inhabits Roatán Island and belongs to lineage 2 of the *M. nigrocinctus* complex [31]. According to our findings, *M. ruatanus* shows a close phylogenetic relationship with *M. nigrocinctus* from Colombia, suggesting possible antigenic similarities between their venom components.

The anticoral-ICP antivenom and anticoral-INS antivenom neutralized the lethal activity of *M. nigrocinctus* venom from Colombia in a preincubation-type murine model. This preclinical assay suggests that treatment with these antivenoms is likely effective in cases of envenomations by this coral snake.

*Micrurus* snakes are generally docile and colorful and are known for their striking combination of red, yellow, and black rings [62]. This appearance can spark curiosity, especially in young children [43,63,64], often placing them among the populations most affected by coral snake bites [64,65,66]. Envenomations by *Micrurus* in children are particularly serious, not only due to the severe neurotoxic syndrome these bites induce, which can lead to rapid respiratory failure [7,17,63], but also due to myotoxic effects, which result in significant increases in plasma creatine kinase levels and are rarely observed in adults [17].

The WHO strategy to decrease the burden of snakebites hinges on four pillars [3]. This research is based on two of these: to empower and engage communities through knowledge about snake biology, snake bite prevention, and management, and to ensure safe and effective treatment through the preclinical evaluation of the antivenom available in Colombia and Costa Rica for the treatment of *M. nigrocintus* envenomation. Adequate knowledge of snake behavior habits and appropriate first aid can reduce the likelihood and consequences of snakebites among people at high risk of encountering them [67]. Considering the distribution of *M. nigrocinctus* in Colombia, the frequency of sightings reported by communities, and the severity of coral snake envenomation in children, the high participation of infant and young population from Apartadó in the workshop “Coral snakes Are Not What People Think They Are” was particularly important, since educating new generations (children and youth) is essential to fostering a new relationship with coral snakes, one that prioritizes conservation, prevention, and sustainable co-existence between humans and snakes.

In addition, this educational approach was implemented with local adult populations and health professionals. Several studies have found that many healthcare workers in snakebite-endemic regions have poor general knowledge about the prevention and management of snakebite envenoming [67,68]. The implemented educational program addressed important issues such as how to recognize the main *Micrurus* species in the region, the differentiation of venomous and non-venomous mimic snakes, how to avoid snakebites, administration of first aid, as well as the importance of seeking immediate medical attention [43,63,69]. Finally, the involvement of communities not only helps prevent coral snake bite, but increases the likelihood that scientists gain access to otherwise difficult-to-find specimens, such as *M. nigrocinctus*. This, in turn, facilitates venom analyses of rare species, the evaluation of antivenoms, and advances the toxinology knowledge for medically significant species.

## 4. Conclusions

This study demonstrates the effectiveness of citizen science and community empowerment as tools to enhance knowledge about *Micrurus nigrocinctus* in Colombia. The involvement of local communities not only aids in the prevention of coral snake bites through education and awareness but also significantly increases the likelihood of accessing rare and elusive species such as *M. nigrocinctus*.

The venom profile of Colombian specimens closely resembles that of Costa Rican populations in terms of protein composition and biological activity and was similarly recognized and neutralized by both anticoral-INS and anticoral-ICP antivenoms, despite underlying phylogenetic divergence. This, in turn, contributes to advancing toxinological knowledge for medically important snakes.

## 5. Materials and Methods

### 5.1. Distribution of M. nigrocinctus in Colombia Based on ‘Citizen Science’ and Scientific Database Reports

Given the elusive nature of *M. nigrocinctus* and the scarcity of records in Colombia, we conducted a detailed assessment of its distribution. We integrated various sources of information on records and occurrences, supplementing the records of localities registered in databases of scientific collections with information from public participation, both from data provided by visited communities and from online databases with verifiable photographic records. Records were reviewed in the main herpetological collections linked to the national system of biological collections of the Alexander von Humboldt Institute for Biological Resources Research through the Biodiversity Information System (BIS) and the Global Biodiversity Information System (GBIF) and in the collection database of the Serpentarium of the University of Antioquia (SUA). Additionally, secondary information available in books, herpetological inventory, characterization reports, reports and information on snakebite accidents, scientific articles, and other academic documents, among others, that may offer information on *M. nigrocinctus* in Colombia, was reviewed.

Citizen science information comes from online databases with verifiable photographic records and from photograph recognition by members of the communities visited. We reviewed photograph records posted in groups created on the Facebook website for this purpose (Aliados de las serpientes—Colombia, Serpientes de Colombia, Identificación de serpientes, Serpientes de Colombia/Fauna ofidica colombiana) and on the biodiversity registry site iNaturaList until February 2025. Keywords such as “*Micrurus nigrocinctus*”, “*nigrocinctus*”, “Coral centroamericana”, and “Coral Urabá” were used. In all cases, only reliable records with accurate taxonomic determinations, clear and precise information on location, non-redundant records, and information consistent with the ecological characteristics of this species were considered. The data were deposited into a database for further analysis.

In addition, to engage communities living in regions with frequent *M. nigrocinctus* sightings, a workshop titled “Coral Snakes Are Not What People Think They Are” was held. This workshop covered key topics such as general information about snakes (both venomous and non-venomous), snakebite prevention (how to avoid bites and what to do in case of an incident), and snake conservation, with emphasis on the ecological and scientific importance of snakes, especially the role venomous species play in antivenom production and medical research. Special attention was given to coral snakes during the training. The workshop featured a mix of vivid photographs, sounds, and videos integrated into an engaging presentation. Hands-on interaction was a key component, with participants using 3D models (including snake skeletons, skulls, and fangs) and real specimens (snake skins, sheds, eggs, and authentic fangs). Participants were allowed to touch a live snake at the end, fostering a more direct connection with these animals.

### 5.2. Molecular and Phylogenetic Analysis of M. nigrocinctus from Colombia

Genomic DNA was extracted from shed skin and blood of two *M. nigrocinctus* specimens from Apartadó and Carepa (Antioquia—Colombia) using E.Z.N.A.^®^ Omega Tissue DNA Kit (Cat. D3396-01) and following the manufacturer’s protocols. The primers, listed in supporting information Supplementary Table S3, allowed us to obtain sequences for two genes (*cytb* and *nd4*). PCR reactions were set up to a final volume of 25 µL, using 1 µL genomic DNA (2 ng/µL), 0.5 µL of each primer (0.2 µM), 2.5 µL of 10X PCR buffer, 0.5 µL total dNTPs (0.2 mM), 0.75 µL of MgCl_2_ (1.5 mM), 0.1 µL of Platinum^®^ Taq DNA Polymerase (1 U), and 19.15 µL of H_2_O. Typical amplification conditions involved initial denaturation at 94 °C for 5 min, followed by 35 cycles with a denaturation step at 95 °C for 45 s, an annealing stage at 55 °C for 45 s, an extension at 72 °C for one min, and a final extension at 72 °C for 10 min. Amplicons were separated by electrophoresis on 1.5% agarose gels in 1X TAE buffer, dyed with GelRed™ Nucleic Acid Gel Stain (Biotium, Inc., Fremont, CA, USA), and visualized under UV light. We performed Sanger sequencing in a capillary automated ABI3500 sequencer (Applied Biosystems^®^, Thermo Scientific; Waltham, MA, USA) at the AUSTRAL omics (Santiago, Chile). The DNA sequences were edited (Trim Ends and de Novo Assemble) and aligned in Geneious Prime v2025.0.3 [70]. For *nd4* and *cytb* genes, the nucleotide sequences were translated into proteins to evaluate the reading frame and ensure the absence of premature stop codons or other nonsense mutations in GeneDoc [71]. Novel sequences were deposited in GeneBank (accession numbers are shown in Supplementary Table S3 and Figure 3).

Data from a total of 41 coral snakes (Supplementary Table S4) were used in the phylogenetic analysis, including *Micruroides euryxanthus* as an outgroup, according to the phylogenetic analysis performed by Jowers et al. [31]. PhyloSuite v 1.2.3 [72] was used for data standardization and concatenation. The CDS genes were aligned using the MACSE algorithm [73] with the vertebrate mitochondrial genetic code and standard code for nuclear genes. We concatenated the *cytb* and *nd4* genes for species with at least two genes sequenced. Bayesian inference (BI) tree was reconstructed using MrBayes 3.2.7 [74], and fitting substitution models were determined in MEGA v 11.0.13 [75] with the Bayesian information criterion (BIC). BI analysis using the default settings by four simultaneous Markov chains was run for five million generations in two independent runs, with sampling every 1000 generations, and the initial 25% of samples were discarded as burn-in. Posterior probabilities were calculated from the consensus of the remaining trees. The confidence of the Bayesian sampling was verified for the free parameters using the effective sample size statistic (ESS) implemented in the software Tracer v.1.5 [76]. All parameters showed ESS greater than 300, and the analyses converged asymptotically, indicating reliable performance.

### 5.3. Characterization of M. nigrocinctus Venom from Colombia and Its Immunorecognition by Commercial Coral Snake Antivenoms

#### 5.3.1. Venoms and Antivenoms

Venom was manually extracted from two *M. nigrocinctus* individuals from the municipalities of Apartadó and Carepa Colombia, located in the department of Antioquia, Colombia. Both specimens were maintained in captivity at the institutional serpentarium of the University of Antioquia, collection Licensee: No. 0524 27 May 2014) and No. 001566 (24 July 2024). A venom pool from Costa Rican specimens of *M. nigrocinctus* was kindly provided by Instituto Clodomiro Picado, University of Costa Rica. Two commercially available equine anticoral antivenoms were evaluated: (a) INS-antivenom (produced by Instituto Nacional de Salud, Colombia; batch N°23AMP01, expiry date November 2025) and ICP-antivenom (produced by Instituto Clodomiro Picado, Costa Rica; batch 7040723ACLQ, expiry date 26 July). Both antivenoms were used before their expiration dates.

#### 5.3.2. Venom Chromatographic and Electrophoretic Profiles

The chromatographic profiles of the venoms of *M. nigrocinctus* from Colombia and Costa Rica were compared. Two mg of each venom were dissolved in 200 µL of 0.1% trifluoroacetic acid (Solution A; TFA), centrifuged at 1250× *g* for 5 min, and fractionated on a C_18_ column (250 × 4.6 mm, 5 µm particle size; Phenomenex. Torrance, CA, USA) using an Agilent 1220 equipment, with monitoring at 215 nm. Elution was performed at a flow rate of 1 mL/min, applying the following gradient toward Solution B (acetonitrile containing 0.1% TFA): 5% B for 5 min, 5–15% B for 10 min, 15–45% B for 60 min, and 45–70% B for 12 min [77]. Also, 30 μg of each venom was analyzed by SDS-PAGE under non-reducing conditions using a 15% gel in a Mini-Protean Tetra Cell electrophoretic system (Bio-Rad, Hercules, CA, USA) at 150 volts. The Precision Plus Protein™ Standards (Broad Range, Bio-Rad, Hercules, CA, USA) were used as molecular weight markers, and the proteins were visualized by Coomassie Blue R-250 staining.

#### 5.3.3. Shotgun Proteomic Profiling

Venom samples of 15 μg of *M. nigrocinctus* from Colombia or Costa Rica, respectively, were concentrated into a single band by SDS-PAGE under reducing conditions after entering the stacking gel. The concentrated bands were visualized with Coomassie R-250 staining, excised from the gel, and subjected to reduction with 10 mM of dithiothreitol for 30 min at 56 °C and alkylation with 50 mM of iodoacetamide for 20 min in the dark, followed by overnight digestion with sequencing-grade trypsin at 37 °C in an automated workstation (Intavis., Waldhäuser, Tübingen, Germany). The resulting peptides were analyzed by nESI-MS/MS using a nano-Easy^®^ 1200 chromatograph in line with a Q-Exactive Plus^®^ mass spectrometer (Thermo Scientific; Waltham, MA, USA). An amount of 5 μL of each digest was loaded onto a C_18_ trap column (75 μm × 2 cm, 3 μm particle size; PepMap, Thermo Scientific; Waltham, MA, USA), washed with 0.1% formic acid (solution A), and separated at a flow rate of 200 nL/min using a C_18_ Easy-spray^®^ column (15 cm × 75 μm, 3 μm particle size). Separation was achieved with a gradient toward solution B (80% acetonitrile, 0.1% formic acid) developed in a total of 120 min (1–5% B in 1 min, 5–26% B in 84 min, 26–80% B in 30 min, 80–99% B in 1 min, and 99% B for 4 min). MS spectra were acquired in positive mode at 1.9 kV, with a capillary temperature of 200 °C, using 1 μscan in the range 400–1600 m/z, maximum injection time of 50 msec, AGC target of 1×10^6^, and resolution of 70,000. The top 10 ions with 2–5 positive charges were fragmented with an AGC target of 3 × 10^6^, minimum AGC 2 × 10^3^, maximum injection time 110 ms, dynamic exclusion time 5 s, and resolution 17,500. MS/MS spectra were processed against protein sequences contained in the Serpentes UniProt/SwissProt database (https://www.uniprot.org/blast, accessed on January 2024) using PEAKS X (Bioinformatics Solutions), and matches were assigned to known protein families by similarity. Cysteine carbamidomethylation was set as a fixed modification, while deamidation of asparagine or glutamine and methionine oxidation were set as variable modifications, allowing up to 3 missed cleavages by trypsin. Parameters for match acceptance were set to FDR < 0.1%, detection of at least 1 unique peptide, and −10lgP protein score ≥ 30. 

#### 5.3.4. Biological Activities of *M. nigrocinctus* Venom from Colombia

The PLA_2_ activity of *M. nigrocinctus* venom from Colombia was tested using the monodisperse synthetic substrate 4-nitro-3-octanoyloxy-benzoic acid (4-NOBA). An amount of 20 μg of venom was dissolved in 25 μL of buffer (10 mM Tris, 10 mM CaCl_2_, 100 mM NaCl, pH 8.0) and added to microplate wells (in triplicate), mixed with 25 μL of the substrate (1 mg/mL in acetonitrile) and 200 μL of the same buffer. After incubation for 60 min at 37 °C, absorbances were recorded at 405 nm using a microplate reader, and activity was expressed as the absorbance change relative to the negative control (substrate alone) [78]. The venom of *M. nigrocinctus* from Costa Rica was compared under the same conditions.

The edema-forming activity was evaluated in the mouse footpad assay [79]. In brief, 5 μg of *M. nigrocinctus* venom from Colombia or Costa Rica, dissolved in 50 μL of saline solution, was injected subcutaneously into the right footpad in groups of three mice of 18–20 g body weight. As a negative control, the left footpad was injected with the same volume of saline solution. The progression of edema was evaluated by measuring the footpad thickness with a caliper at 0.5, 1, 2, and 4 h after the injection.

The myotoxicity activity was evaluated using a group of three mice that received an intramuscular injection containing 10 μg of *M. nigrocinctus* venom from Colombia or Costa Rica (in 50 μL Buffer PBS) into the gastrocnemius. Control mice received an injection of PBS. Blood was collected after 1.5 h from the tip of the tail into heparinized capillaries, and the activity of creatine kinase (CK) in plasma was determined using a UV kinetic assay (CK-Nac, Wiener) [80].

To evaluate the lethal activity, various amounts of *M. nigrocinctus* venom from Colombia (from 3.5 to 56 µg), dissolved in 250 µL of saline solution, were injected intraperitoneally (i.p.) in groups of four mice (16–18 g of body weight). Deaths were recorded after a 48 h observation period, and the median lethal dose (LD_50_) was calculated by probits [81]. Animal experiments were conducted under a study protocol approved by the Institutional Committee for the Care and Use of Laboratory Animals (CICUA) from the University of Antioquia (License No. 160 of 2024).

#### 5.3.5. Venom Immunorecognition and Neutralization by Commercial Anticoral Antivenoms

The antibody titers of the ICP-antivenom and INS-antivenom, respectively, were assessed against whole *M. nigrocinctus* venoms from Colombia and Costa Rica using an enzyme-linked immunosorbent assay (ELISA). Each microplate well was coated with 0.1 μg of complete venom diluted in 100 μL of coating buffer (0.1 M Tris, 0.15 M NaCl, pH 9.0) and incubated overnight at room temperature. The wells were blocked with 100 μL of 1% bovine serum albumin in phosphate buffer (BSA-PBS; 0.04 M phosphates, 0.12 M NaCl, pH 7.2) for 90 min. Serial dilutions of each antivenom or a non-immune equine serum as a negative control (1:500 to 1:1.093.500) were added to the wells and incubated for 90 min at room temperature. After washing, a peroxidase-labeled anti-horse IgG conjugate (1:8000; Sigma-Aldrich., Darmstadt, Germany) was added as the secondary antibody and incubated for 90 min at room temperature. Following a final wash, 100 μL of peroxidase substrate (2 mg/mL of *o*-phenylendiamine diluted in 0.1 M sodium citrate, pH 5.0; 4 μL of 30% H_2_O_2_ per 10 mL of final solution) was added for color development. The absorbance was measured at 492 nm using a Multiskan Sky spectrophotometer (Thermo Scientific; Waltham, MA, USA).

The ability of commercial equine antivenoms to neutralize the lethal effect of *M. nigrocinctus* was evaluated by preincubation experiments. Groups of four mice (16–18 g of body weight) were injected intra-peritoneally (i.p.) with 250 μL of a solution containing 25.8 μg of *M. nigrocinctus* venom (equivalent to a challenge of 3 × LD_50_), previously incubated for 30 min at 37 °C with the antivenoms (ICP or INS) in a proportion of 0.2 mg of venom per mL of antivenom. A group of control mice received the same dose of venom and were incubated only with saline solution. Deaths were recorded after 48 h.

## Figures and Tables

**Table 1:** The preincubation neutralizing ability of anticoral-INS antivenom and anticoral-ICP antivenom on the lethal effect of *M. nigrocinctus* venom from Colombia in mice.

Group	Venom/Antivenom	Dead/Inject ^1^
*M. nigrocinctus* venom	0	4/4
*M. nigrocinctus* venom + anticoral-ICP	0.2 mg/mL	0/4
*M. nigrocinctus* venom + anticoral-INS	0.2 mg/mL	1/4

^1^ The lethality-neutralizing ability of antivenom was evaluated by preincubating with the venom at 37 °C for 30 min and then injecting the mixture into mice (16–18 g of body weight) by i.p. route. Deaths were recorded at 48 h.

## Data Availability

The original contributions presented in this study are included in this article and Supplementary Material. Further inquiries can be directed to the corresponding author.

## Supplementary Materials

The following supporting information can be downloaded at: https://www.mdpi.com/article/10.3390/toxins17060268/s1, Figure S1: *M. nigrocinctus* from Córdoba. Individual deposited in the collection of the Serpentarium of the University of Antioquia—SUA #1930; Table S1: Database of distribution of *M. nigrocinctus* based on “citizen science” and scientific database reports; Table S2: Protein matches obtained by nESI-MS/MS shotgun analysis of tryptic peptides derived from *Micrurus nigrocinctus* (Colombia) whole venom; Table S3. Primers and accession numbers for the new sequences deposited in GenBank; Table S4. Species, countries, localities, and GenBank accession numbers used in this study. The order of the species corresponds from top to bottom in the phylogenetic tree.
