# Supplementary material for: Micrurus nigrocinctus in Colombia: Integrating Venomics Research, Citizen Science, and Community Empowerment
Source: Toxins (Basel). 2025 May 27;17(6):268. doi: 10.3390/toxins17060268 (PMC12197593; doi:10.3390/toxins17060268)

M. nigrocinctus from Córdoba

Individual deposited in the collection of the Serpentarium of the University of Antioquia - SUA #1930

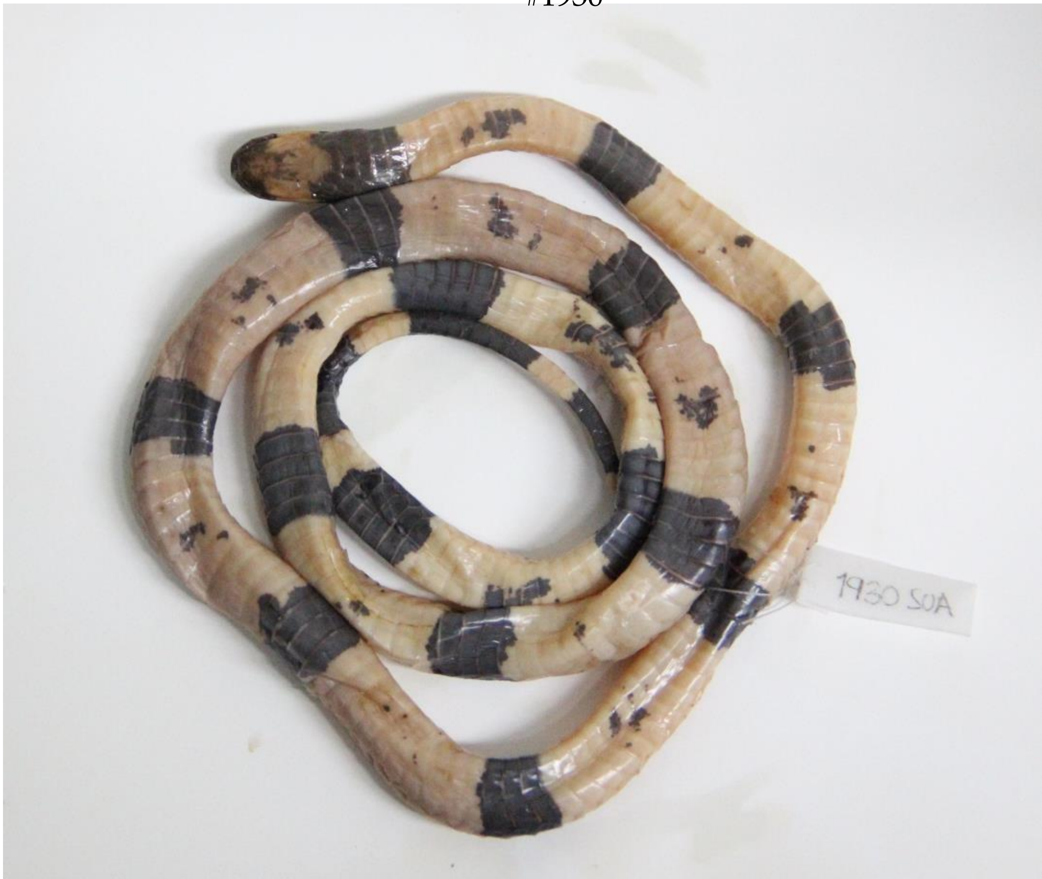

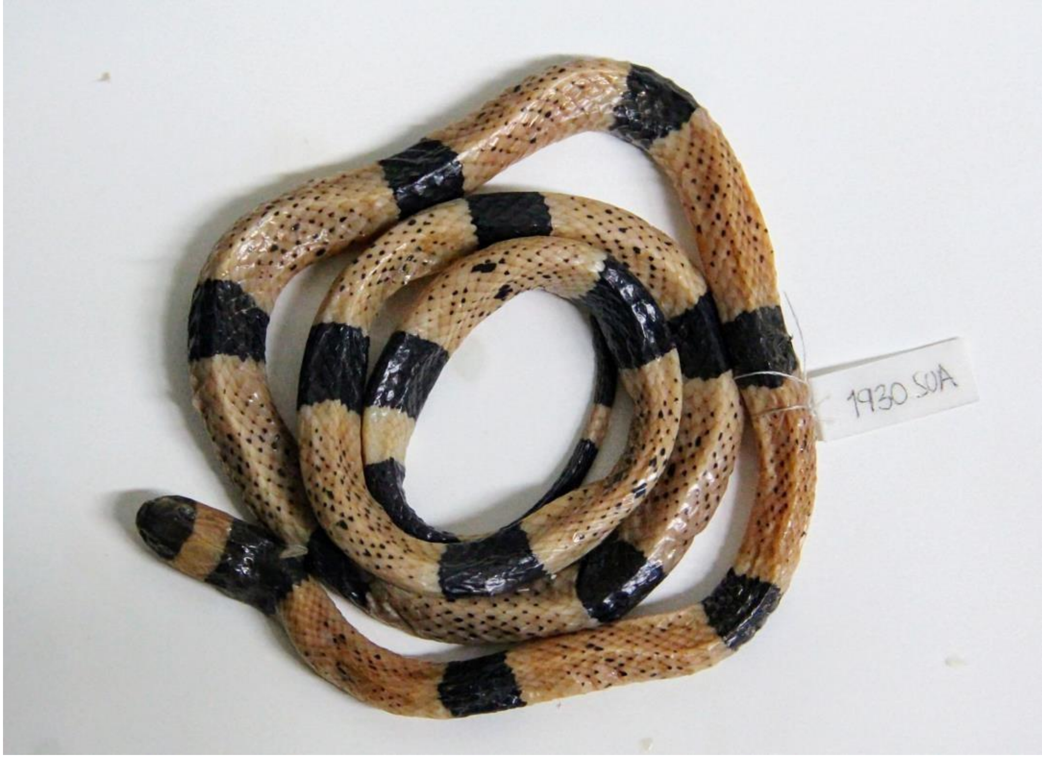

Supplement: Supplementary file 1 [file toxins-17-00268-s001.zip › toxins-3612957-supplementary (1)/Figure S1_M. nigrocinctus_Cordoba (1).pdf]
